# Jiawei Danggui Buxue Decoction Reduces Apoptosis and EMT of Renal Interstitial Fibrosis by Regulating JAK2/STAT3 Signaling Pathway

**DOI:** 10.2174/0113862073355322250226050458

**Published:** 2025-03-11

**Authors:** Xin Jiang, Yinghang Wang, Saiyue Qiu, Lu Tang, Meixiu Luo, Zhi Pan

**Affiliations:** 1 College of Integrative Medicine, Changchun University of Chinese Medicine, Changchun, 130117, China;; ^2^ The Affiliated Hospital to Changchun University of Chinese Medicine, Changchun, 130021, China;; 3 Jilin Ginseng Academy, Changchun University of Chinese Medicine, Changchun, 130117, China

**Keywords:** Renal interstitial fibrosis, JAK2/STAT3 signaling pathway, EMT, cell apoptosis, ECM, JDBD

## Abstract

**Background:**

Renal interstitial fibrosis (RIF) is the primary pathological progression in chronic kidney disease (CKD). Given the constraints related to cost and adverse effects of current treatments, it is crucial to explore novel and efficacious therapeutic strategies. The purpose of this study was to elucidate the potential of Jiawei Danggui Buxue Decoction (JDBD) to reduce apoptosis and epithelial-mesenchymal transition (EMT) in RIF by regulating the Janus kinase 2 (JAK2)/Signal Transducer and Activator of Transcription 3 (STAT3) pathway.

**Methods:**

An angiotensin II (Ang II)-induced HK-2 cells model and a unilateral ureteral obstruction (UUO) animal model were employed to replicate the RIF model. A total of 48 male Wistar rats (weighing 200-220g) were acclimated for 1 week and then randomly divided into 6 groups (sham operation, UUO, Losartan potassium tablets, and three JDBD dosage groups: high, medium, and low, n=8). After the acclimatization period, UUO models were established in 40 rats through surgery, excluding the sham operation group. Each group received the corresponding drug *via* gavage for 2 weeks. After 2 weeks, rats were anesthetized, and tissues were collected for subsequent analysis. Renal function tests and histological stains were used to evaluate renal damage and histopathological alterations in rats. Cell viability was examined using the CCK-8 assay. Apoptosis was identified through the utilization of flow cytometry and assessment of mitochondrial membrane potential, along with other techniques. We identified and examined the expression of EMT and extracellular matrix (ECM)-related factors, as well as the JAK2/STAT3 pathway.

**Results:**

*In vivo* experiments indicated that JDBD effectively reduced renal dysfunction in UUO rats, ameliorated pathological changes in renal tissues, and significantly modulated the JAK2/STAT3 signaling pathway to inhibit EMT and apoptosis, thereby reducing ECM deposition. Furthermore, JDBD markedly increased the survival rate of Ang II-treated HK-2 cells and reduced apoptosis. The *in vitro* experimental results further confirmed that JDBD ameliorates RIF by regulating the JAK2/STAT3 pathway.

**Conclusion:**

JDBD exhibits anti-apoptotic and EMT-inhibiting functions in RIF, potentially mediated by targeting and inhibiting JAK2/STAT3 signaling transduction.

## 1 INTRODUCTION

Approximately 9.1% of humanity is affected by Chronic Kidney Disease (CKD), which is a substantial international medical challenge [1]. Renal interstitial fibrosis (RIF), a fundamental pathological process in CKD, is defined by the proliferation of extracellular matrix (ECM), epithelial-mesenchymal transition (EMT), fibroblast activation and proliferation, inflammatory responses, apoptosis, and microvascular rarefaction [2]. Notably, the ECM composition in RIF undergoes substantial alterations, especially in the expression and deposition of collagen types I and III (Col-1 and Col-3) and fibronectin (FN), which are critical to fibrosis development [3]. During the EMT process, renal tubular epithelial cells (RTECs) differentiate into interstitial cells, migrating into the renal interstitium, where they significantly contribute to ECM production, thereby exacerbating fibrosis [4]. Additionally, persistent apoptosis promotes the accumulation of the ECM, which contributes to the acceleration of RIF [5]. Consequently, an in-depth understanding of RIF mechanisms is essential for preventing and mitigating CKD progression.

The JAK2/STAT3 signaling pathway is a ubiquitous and vital signal transduction pathway in organisms [6]. It is activated during RIF, leading to the regulation of EMT and apoptosis in RTECs and thereby exacerbating RIF [7, 8]. Jiawei Danggui Buxue Decoction (JDBD), composed of *Astragalus membranaceus* (Fisch.) Bunge, *Angelica sinensis* (Oliv.) Diels, and *Panax ginseng* C. A. Mey. serve crucial functions in immune modulation and organ protection. These herbs demonstrate diverse physiological traits, including anti-fibrotic, anti-inflammatory, anti-apoptotic, and antioxidant actions [9-11]. Danggui Buxue Decoction (DBD), derived from Li Dongyuan’s “The theory of internal and external injury discrimination,” has been integral to Chinese medical heritage for millennia, noted for its ability to supplement Qi and generate blood. This formula has yielded favorable therapeutic results in CKD patients [12]. Further studies have indicated that both DBD and *Panax ginseng* regulate the TGF-β/Smad signaling pathway to ameliorate RIF [13, 14]. Consequently, we have augmented DBD with additional *Panax ginseng* to delve deeper into the specific mechanisms through which JDBD can prevent and manage RIF.

This study aimed to clarify the mechanism by which JDBD treatments improve RIF. The method likely involves suppressing the JAK2/STAT3 pathway, which in turn weakens the EMT process, decreases apoptosis, and reduces ECM deposition. These findings offer both theoretical and experimental evidence to support the possible use of JDBD in clinical settings.

## 2 MATERIALS AND METHODS

### 2.1 JDBD Preparation

The medicinal ingredients in the JDBD are *Astragalus membranaceus*, *Angelica sinensis*, and *Panax ginseng*, purchased from Jilin Province Beiyao Traditional Chinese Medicine Pharmaceutical Group Co., Ltd. According to the Chinese Pharmacopoeia, the clinical dosages are 30 g of *Astragalus membranaceus*, 6 g of *Angelica sinensis*, and 6 g of *Panax ginseng*. Using the equivalent dose ratios between humans and animals provided in Pharmacological Experimental Methodology, the equivalent dose for rats was calculated to be 4.4 g/kg, which was designated as the JDBD medium dose (JDBD-M) group. The JDBD low dose (JDBD-L) was set at 2.2 g/kg, and the high dose (JDBD-H) at 8.8 g/kg. *Astragalus membranaceus*, *Angelica sinensis*, and *Panax ginseng* were soaked in distilled water in a 5:1:1 ratio for 1 h, then decocted with tenfold distilled water on high heat for 45 min, followed by filtration. The mixture was then decocted again on low heat for 25 min after adding tenfold distilled water and filtered again. After the two filtrates were combined and concentrated, the final concentrations were 0.22 g/mL, 0.44 g/mL, and 0.88 g/mL. These were then kept for later use at 4 °C.

### 2.2 Animal and Cell Culture

Specific pathogen-free (SPF) 84 male Wistar rats (200g-220g) were purchased from Changchun Yisi Experimental Animal Technology Co., Ltd. (License No. : SCXK (Ji) 2020-0002).

A total of 84 male Wistar rats, with a weight range of 200-220 g, were chosen for the animal trials. The Animal Experimental Ethics Committee at Changchun University of Traditional Chinese Medicine accepted this animal study (Approval No.: 2022542). During a one-week acclimation period, the rats resided in a controlled environment with a consistent temperature of 21±2 °C and a humidity range of 45-65%. The rats were exposed to a 12 h cycle of light and darkness.

HK-2 cells were grown in DMEM/F12 medium supplemented with 10% FBS (Hyclone). HK-2 cell line was purchased from Shanghai Fuheng Biotechnology Co., Ltd. and cultured in the DMEM/F12 medium supplemented with 10% FBS (Hyclone). The cells were incubated at 37 °C and 5% CO_2_ in an incubator.

### 2.3 Preparation of Drug-containing Serum

The preparation of the drug-containing serum was conducted as follows: 36 rats were randomly allocated into 5 groups. Twelve rats constituted the normal group, with serum from this group used to treat the cells in both the normal and AngII groups. The remaining 24 rats were equally divided among the other 4 groups: Losartan potassium tablets (LPT) group (10.5 mg/kg), JDBD-H group (8.8 g/kg), JDBD-M group (4.4 g/kg), and JDBD-L group (2.2 g/kg). Rats in each group received varying concentrations of LPT and JDBD decoction *via* gavage at a volume of 1 mL/100 g. Rats in the normal group were administered an equivalent quantity of distilled water *via* gavage. Rats in each group were intragastrically administered once a day. The rats were anaesthetized with a 20% urethane injection 1 h post-administration on the fourth day, and blood samples were obtained from the abdominal aorta. The serum was inactivated and sterilised following centrifugation, and it was subsequently stored at -20 °C.

### 2.4 Establishment of Animal Model and Treatment

Forty-eight rats were assigned randomly to six groups (sham operation group, unilateral ureteral obstruction (UUO) group, LPT (10.5 mg/kg) group, JDBD-H (8.8 g/kg) group, JDBD-M (4.4 g/kg) group, JDBD-L (2.2 g/kg) group, n=8). The rats were anaesthetized with 20% urethane for either UUO or sham operations after fasting for 12 hours prior to surgery. After being positioned supinely, the preoperative skin was prepared and disinfected. Except for the sham operation group, a longitudinal cut was made near the side of the left kidney in the other groups, and the proximal end of the left ureter was ligated at two locations [15]. The ureter was solely dissected by the sham operation group without being ligated. The muscle layer and skin were sutured in turn, and 8 × 10^4^ units of penicillin were injected every day for the first 3 days after surgery to prevent infection [16]. From the first day post-operation, the LPT group and various JDBD groups were administered LPT (10.5 mg/kg) and corresponding dosages of JDBD daily by gavage. The sham operation and UUO groups were both given an equal amount of distilled water. Throughout the feeding period, the rats' body weight and health state were closely observed daily. On the 14th day post-operation, after fasting for 12 h, the rats were administered urethane by intraperitoneal injection to induce anaesthesia, and kidney tissues and blood samples were subsequently obtained [17]. In order to obtain serum, the blood was centrifuged at a speed of 3000 rpm for 15 min, and the weights of the left and right kidneys were recorded. The left kidney was divided into two equal sections along the transverse section: one portion was immersed in a 4% paraformaldehyde solution for fixation, while the other part was frozen at a temperature of -80 °C.

### 2.5 Biochemical Assays in Serum

The levels of serum creatinine (Scr) and blood urea nitrogen (BUN) were determined using an automated biochemical analyzer (Chemray 800, Leidu, China).

### 2.6 H&E and Masson Staining

Kidney tissues were fixed, dehydrated, embedded, sectioned at 4 µm thickness, dried, dewaxed, and stained with hematoxylin and eosin (H&E) (G1120; Solaibao, China) as well as Masson's trichrome (G1340; Solaibao, China), then mounted on slides and sealed [18]. Five random fields were selected for observation under a 40 × light microscope. The degree of renal injury was evaluated by assigning scores to the identified histological changes in renal tubules and glomeruli. The evaluation of histopathological changes was based on a scale of 0-4, 0: normal without injury, 1: the degree of injury was 0-25%, 2: the degree of injury was 25-50%, 3: the degree of injury was 50-75%, 4: the degree of injury was more than 75% [19]. RIF was quantified by measuring the area of Masson's trichrome staining and calculating the percentage of fibrosis. ImageJ software was utilized to analyze the average histopathological change scores and fibrosis percentages in five randomly selected fields for each group at 400 × magnification [20].

### 2.7 Cell Viability Assay

100 μL of medium was used to seed HK-2 cells at a density of 1 × 10^4^ cells/well into 96-well plates. Each experimental group consisted of 6 replicate wells across 6 different groups. Cells were treated with the respective drugs detailed in Table **1**. 10 μL of CCK-8 solution (C0037; Beyotime, China) was added to each well as a supplement after 48 h of incubation. The OD at 450 nm was measured using a microplate reader (RT-6000, Leidu, China) after 1 h of incubation. The cell viability was calculated using the formula: (OD_sample_-OD_blank_ / OD_normal_-OD_blank_) × 100% [21].

### 2.8 Flow Cytometry Detection of Apoptosis

On 6-well plates, HK-2 cells were added at a cell count of 2 × 10^5^ cells/well. Following their adhesion, they were exposed to different drug‐containing serum, as listed in Table **1**, for 48 h. The cells were then digested, washed, and counted, and 10^5^ cells were collected from each sample. The above steps were repeated, with three samples taken from each group. Next, the cells were re-suspended with 195 μL of binding solution, then 5 μL of Annexin V-FITC, and 10 μL of propidium iodide dye solution. After the cells were well combined, they were incubated for 20 min at room temperature. Fluorescence was detected by flow cytometry, and the resulting data were analyzed using CFlow software [22].

### 2.9 Determination of Mitochondrial Membrane Potential by JC-1 Fluorescent Probe

On 6-well plates, HK-2 cells were added at a cell count of 1 × 10^5^ cells/well and treated as described in Table **1** for 48 h. We treated each well with 1 mL of JC-1 probe staining solution (C2003S; Beyotime, China) and incubated it at 37 °C for 30 min. We then washed the sample with JC-1 buffer. 2 mL of DMEM/F12 complete media was then put into each well, and pictures were taken using a fluorescent microscope (Bimu Instrument, China) [23].

### 2.10 Detection of Caspase-3 Activity in Living GreenNuc^TM^ Cells

HK-2 cells were seeded at a count of 1 × 10^5^ cells/well in 6-well plates and 1 × 10^4^ cells/well in 96-well plates. After 48 h of treatment, as detailed in Table **1**, procedures were conducted in accordance with the GreenNuc™ Live Cell Caspase-3 Activity Assay Kit (C1115; Beyotime, China) instructions. Fluorescence microscopy was employed for cellular observation and imaging, while a microplate reader assessed the OD values [24].

### 2.11 Hoechst 33342 Test of Apoptosis

The 6-well plates described in section 2.10 were stained following the GreenNuc™ Live Cell Caspase-3 Activity Assay Kit instructions, and the staining solution was aspirated. Each well was treated with 1 mL of Hoechst 33342 staining solution (C1025; Beyotime, China) and incubated at 37 °C for a duration of 15 min. Finally, the cells underwent a washing process, and 1 mL of PBS was added to each well, and images were photographed with a fluorescence microscope [25].

### 2.12 Enzyme-linked Immunosorbent Assay (ELISA)

Rat serum and HK-2 cell supernatant treated for 48 h, as described in Table **1**, were analyzed using ELISA assays (Sino Best, China) following the directions for use provided in the respective ELISA kits to measure the levels of Col-1, Col-3, and FN.

### 2.13 Quantitative Polymerase Chain Reaction (qPCR)

Primers for B-cell lymphoma-2 (Bcl-2), Bcl-2 Associated X (Bax), Caspase-3, E-cadherin, α-smooth muscle actin (α-SMA), and β-actin were supplied by Shanghai Sangon Biological and Technological Company. Kidney tissues and cells were subjected to RNA extraction using the RNAeasy Total RNA Kit (Tiangen Biotech, China). The RNA concentration and purity were evaluated using a NanoPhotometer & Spectrophotometer (IMPLEN, USA). The isolated RNA was then converted into cDNA, following the methods supplied. A 10 μL qPCR reaction mixture was prepared, consisting of 0.5 μL of each primer, 0.8 μL of target DNA, 5 μL of TB Green Premix Ex Taq (Tli RNaseH Plus) (Takara, Japan), and nuclease-free water to a final volume of 10 μL. Amplification was conducted using a fluorescent qPCR instrument. The target genes' expression levels were evaluated utilizing the 2^-ΔΔCt^ approach, with β-actin as the internal reference gene. Primer sequences are shown in Table **2**. All experiments were repeated 3 times.

### 2.14 Western Blot

Renal tissue and HK-2 cells were lysed by adding protease and phosphatase inhibitors to RIPA buffer to extract total protein. The BCA method was employed to determine the protein concentration. The same quantity of protein was transferred to the PVDF membrane following SDS-PAGE. The membrane was blocked, washed and incubated with primary antibodies against E-cadherin (1:1200), α-SMA (1:800), JAK2 (1:500), p-JAK2 (1:800), STAT3 (1:1200), p-STAT3 (1:800) and GAPDH (1:1000) overnight at 4 °C. After washing, the membranes were incubated with HRP-conjugated anti-rabbit IgG antibody (1:1000) for 1 h, followed by washes. Protein bands were detected through enhanced chemiluminescence and visualized with a gel imaging system (T5200, Tanon, China). All experiments were repeated 3 times, and Image J software was employed for image quantification.

### 2.15 Statistical Analysis

The data were reported as the mean ± standard deviation and analysed using GraphPad Prism version 9.5 The statistical significance of variations in mean values among groups was evaluated using a one-way analysis of variance (ANOVA), followed by Tukey's multiple comparison test. A p-value less than or equal to 0.05 was deemed statistically significant.

## 3 RESULTS

### 3.1 *In Vivo* Renoprotective Effect

#### 3.1.1 JDBD Ameliorates Renal Morphology and Renal Function in UUO Rats

**Fig. (1):**
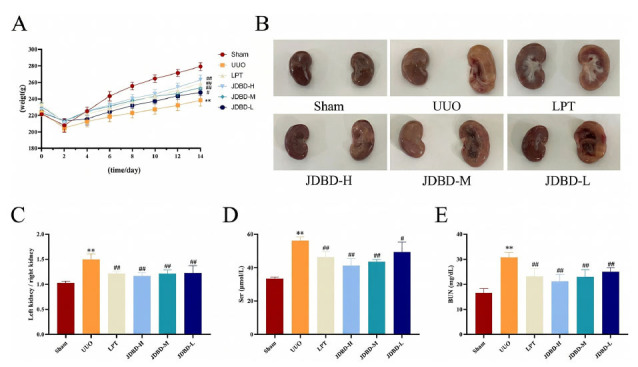
Protective effects of JDBD on the kidneys of UUO rats. **(A)** Changes in rat body weight for 14 days. **(B)** Illustrations depicting blocked kidneys in different groups. **(C)** Weight ratio between the left and right kidneys. **(D)** Scr levels. **(E)** BUN levels. Data are presented as mean ± SD (*n* = 6). ** *P*< 0.01 *vs.* sham group; ^##^ *P* < 0.01, ^#^ *P* < 0.05 *vs.* UUO group.

Throughout the medication period, daily observations were made of the health status and body weight of the rats. All groups experienced an increase in body weight 14 days post-operation; however, the rise in the UUO group was considerably smaller in comparison to that reported in the sham operation group (*P* < 0.01), while the LPT group and various JDBD dose groups showed greater increases compared to the UUO group (*P* < 0.05 and *P* < 0.01) (Fig. **1A**). Fourteen days following the gavage, the rats' kidneys were harvested. In the UUO group, the left kidney was significantly larger than the right, exhibiting a rough surface, color changes, and considerable effusion. Renal changes in the LPT group and various JDBD dosage groups were less severe compared with the UUO group (Fig. **1B**). The left-to-right kidney weight ratio had been greater in the UUO group than in the sham operation group (*P* < 0.01). Conversely, it was lower in the LPT group and various JDBD dosage groups compared with the UUO group (*P* < 0.01) (Fig. **1C**).

Furthermore, Scr and BUN levels were measured. The Scr and BUN levels in the UUO group were considerably higher than those in the sham operation group (*P* < 0.01). Conversely, LPT and JDBD groups could reverse the increase in Scr and BUN (*P* < 0.05 and *P* < 0.01) (Figs. **1D**, **E**). JDBD exerts a protective effect on renal function.

#### 3.1.2 JDBD Reduced Renal Injury and Interstitial Fibrosis

**Fig. (2):**
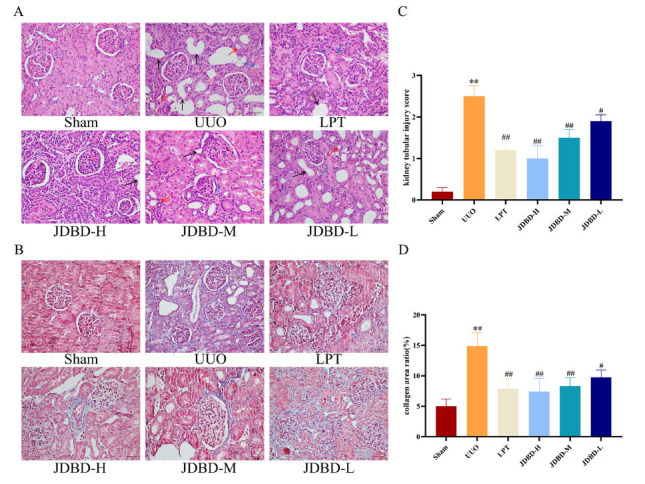
JDBD alleviates kidney injury in UUO rats. **(A)** Representative kidney H&E staining images from each group (400 × magnification). Black arrow: renal tubular dilatation; red arrow: renal tubular atrophy; blue arrow: renal tubular edema. **(B)** Representative Masson's trichrome staining images from each group (400 × magnification). **(C)** Comparison of renal tubular injury scores based on H&E staining. **(D)** Comparison of collagen area ratios based on Masson staining. Data are presented as mean ± SD (*n* = 3). ** *P* < 0.01 *vs.* sham group; ^##^ *P* < 0.01, ^#^ *P* < 0.05 *vs.* UUO group.

H&E and Masson's trichrome staining were employed to conduct pathological analyses of rat renal tissue sections. As shown in Fig. (**2A**), the renal tubules in the sham operation group appeared normal, with orderly-arranged, undamaged epithelial cells. Conversely, the UUO group, as well as those treated with LPT and various doses of JDBD, demonstrated significant renal alterations, including dilation of renal tubules, interstitial edema, formation of tubular casts, vacuolar degeneration of epithelial cells, infiltration of inflammatory cells, and renal tubule atrophy. Renal damage was alleviated following intervention with LPT and JDBD. Masson's trichrome staining revealed extensive collagen fibers in the UUO group compared to the sham operation group. Notably, the collagen fiber area in the LPT and various doses of the JDBD groups was significantly reduced compared to the UUO group (Fig. **2B**). The renal tubular damage scores and the percentage area of collagen-positive proteins (Figs. **2C**, **D**) demonstrated that the aforementioned differences were statistically significant (*P* < 0.05 and *P* < 0.01).

#### 3.1.3 JDBD Reduced ECM Deposition and EMT in UUO Rats

**Fig. (3):**
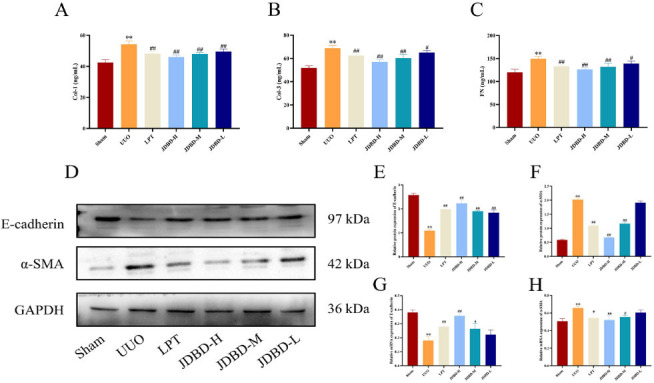
JDBD reduced ECM deposition and EMT in UUO rats. **(A-C)** The levels of Col-1, Col-3 and FN in serum were measured by ELISA. **(D-F)** Western blot analysis showed the levels of E-cadherin and α-SMA protein in the renal tissue of rats. **(G, H)** qPCR analysis showed the levels of E-cadherin and α-SMA mRNA in the renal tissue of rats. Data are presented as mean ± SD (*n* = 3). ** *P* < 0.01 *vs.* sham group; ^##^ *P* < 0.01, ^#^ *P* < 0.05 *vs.* UUO group.

The predominant feature of RIF is the accumulation of ECM. We assessed the influence of JDBD on renal ECM deposition by measuring serum levels of Col-1, Col-3, and FN in rats. The UUO group exhibited substantially elevated levels of Col-1, Col-3, and FN in comparison to the sham operation group (*P* < 0.01). Nevertheless, treatment with JDBD and LPT markedly inhibited these changes (*P* < 0.05 and *P* < 0.01) (Figs. **3A**-**C**).

EMT was assessed by measuring the expression of E-cadherin and α-SMA. The UUO group exhibited a decrease in E-cadherin and an increase in α-SMA protein and mRNA expression in comparison to the sham operation group (*P* < 0.01). As illustrated in Figs. (**3D**-**H**), treatment with JDBD and LPT resulted in an increase in E-cadherin and a decrease in α-SMA protein and mRNA expression (*P* < 0.05 and *P* < 0.01).

In conclusion, JDBD effectively mitigates the severity of RIF by reducing ECM deposition and attenuating EMT transition.

#### 3.1.4 JDBD Inhibits Renal Cell Apoptosis in UUO Rats

**Fig. (4):**
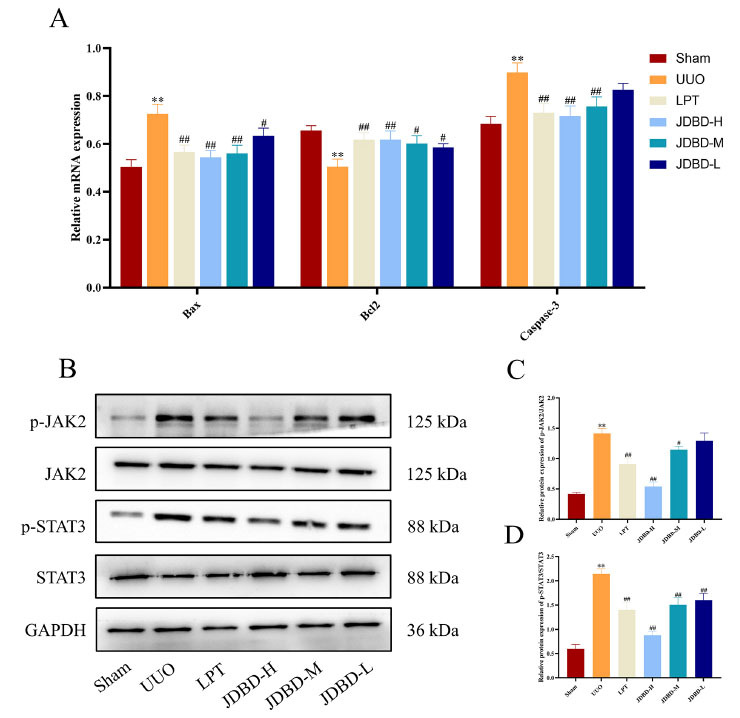
JDBD inhibits apoptosis and regulates the JAK2/STAT3 signaling pathway *in vivo*. **(A)** qPCR analysis showed the levels of Bax, Bcl-2 and Caspase-3 mRNA in the renal tissue of rats. **(B-D)** Western blot analysis showed the levels of p-JAK2/JAK2 and p-STAT3/STAT3 protein in the renal tissue of rats. Data are presented as mean ± SD (n = 3). ** *P*< 0.01 *vs.* sham group; ^##^ *P* < 0.01, ^#^ *P* < 0.05 *vs.* UUO group.

We evaluated the mRNA expression levels of Bax, Bcl-2, and Caspase-3 to verify the anti-apoptotic effects of JDBD *in vivo*. The UUO group exhibited an increase in Bax and Caspase-3 mRNA expression, while Bcl-2 mRNA expression was decreased compared to the sham operation group (*P* < 0.01). After being treated with JDBD and LPT, there was a decline in the levels of Bax and Caspase-3 mRNA and an increase in Bcl-2 mRNA expression (*P* < 0.05 and *P* < 0.01) (Fig. **4A**). These data indicate that JDBD significantly inhibits renal cell apoptosis in UUO rats.

#### 3.1.5 The Effect of JDBD on the Expression of the JAK2/STAT3 Signaling Pathway in UUO Rats

Protein levels of JAK2, p-JAK2, STAT3, and p-STAT3 were quantified *via* Western blot analysis. Compared to the sham operation group, the UUO group exhibited elevated ratios of p-JAK2/JAK2 and p-STAT3/STAT3 (*P* < 0.01). Subsequent treatment with JDBD and LPT significantly reduced these ratios (*P* < 0.05 and *P* < 0.01), as shown in Figs. (**4B**-**D**). These data suggest that JDBD may modulate cellular apoptosis and EMT, contributing to the improvement of RIF by regulating the JAK2/STAT3 signaling pathway.

### 3.2 *In Vitro* Renoprotective Effect

#### 3.2.1 The Effect of JDBD-containing Serum on the Viability of HK-2 Cells Induced by AngII

The cell viability experiments, depicted in Fig. (**5A**), revealed a substantial decrease in cell viability in the AngII group compared to the normal group (*P* < 0.01). Conversely, treatment with both LPT and various doses of JDBD significantly enhanced cell viability compared to the model group (*P* < 0.01), indicating that JDBD effectively enhances the vitality of HK-2 cells.

#### 3.2.2 JDBD-containing Serum Can Reduce AngII-induced ECM Deposition and EMT in HK-2 Cells

To assess the impact of JDBD-containing serum on ECM deposition and EMT in HK-2 cells induced by AngII, we measured the levels of Col-1, Col-3, and FN using ELISA. Additionally, the expression of α-SMA and E-cadherin at the protein and mRNA levels was assessed *via* Western blot and qPCR, respectively. As illustrated in Figs. (**5B**-**D**), the AngII group exhibited notably greater amounts of Col-1, Col-3, and FN compared to the normal group (*P* < 0.01). Subsequently, treatment with JDBD and LPT resulted in a marked decrease in these levels (*P* < 0.05 and *P* < 0.01). The AngII group also exhibited significantly higher protein and mRNA expression levels of α-SMA, while E-cadherin levels were lower (*P* < 0.01). After treatment, the expression of α-SMA was attenuated and E-cadherin was increased in both protein and mRNA forms (*P* < 0.05 and *P* < 0.01), as shown in Figs. (**5E**-**I**). These results demonstrate that serum containing JDBD effectively reduces ECM deposition and reverses EMT induced by AngII in HK-2 cells.

#### 3.2.3 JDBD-containing Serum Inhibits AngII-induced Apoptosis of HK-2 Cells

**Fig. (6):**
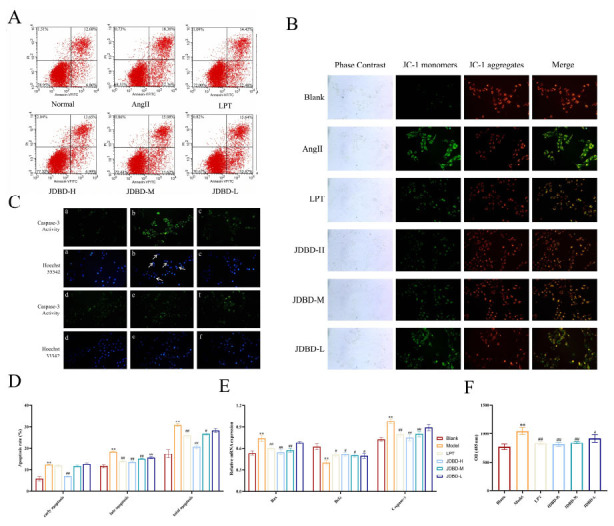
JDBD-containing serum inhibits AngII-induced apoptosis of HK-2 cells. **(A)** JDBD-containing serum inhibited HK-2 cell apoptosis induced by AngII. **(B)** Representative images of JC-1 staining (200 × magnification). **(C)** Fluorescence intensity and morphological changes of apoptosis were observed using Caspase-3 activity assays and Hoechst 33342 staining (200 × magnification). a: normal group, b: AngII group, c: LPT group, d: JDBD-H group, e: JDBD-M group, and f: JDBD-L group. **(D)** Statistical analysis of HK-2 cell apoptosis rate. **(E)** qPCR analysis showed the levels of Bax, Bcl-2 and Caspase-3 mRNA in HK-2 cells. **(F)** the level of Caspase-3 activity. Data are presented as mean ± SD (*n* = 3). ** *P*< 0.01 *vs.* normal group; ^##^ *P*< 0.01, ^#^ *P*< 0.05 *vs.* AngII group.

We compared the anti-apoptotic effects of JDBD and LPT in HK-2 cells. As shown in Figs. (**6A**, **D**), flow cytometry results indicated that the early, late, and total apoptosis rates in the AngII group were significantly higher than those in the normal group (*P* < 0.01). JDBD reversed early apoptosis (*P* < 0.01), and both JDBD and LPT reduced the number of late apoptotic cells (*P* < 0.01), thereby decreasing the total apoptosis rate (*P* < 0.01, *P* < 0.05). We measured the expression of apoptosis-related mRNA, which showed a decrease in the expression of pro-apoptotic Bax and Caspase-3 mRNA, alongside an increase in the expression of anti-apoptotic Bcl-2 mRNA in HK-2 cells treated with LPT-containing serum and JDBD-containing serum following AngII induction (*P* < 0.05 and *P* < 0.01) (Fig. **6E**). Further, Caspase-3 activity assays confirmed that JDBD-containing serum could inhibit Caspase-3 activity (*P* < 0.05 and *P* < 0.01) (Figs. **6C**, **F**). In addition, Hoechst 33342 staining was used to observe that compared with the normal group, a large number of nuclear condensation and division were observed in the AngII group, which proved that apoptosis occurred. After JDBD and LPT intervention, apoptosis was reduced (Fig. **6C**).

Furthermore, JC-1 fluorescent dye was employed to assess mitochondrial membrane potential. Upon elevation of the membrane potential, JC-1 aggregates and emits red fluorescence. Conversely, a reduction in membrane potential leads to the emission of green fluorescence by JC-1. The results indicate that treatment with JDBD induces an increase in mitochondrial membrane potential in HK-2 cells, as evidenced by reduced green fluorescence, suggesting that JDBD inhibits apoptosis in these cells (Fig. **6B**).

#### 3.2.4 The Effect of JDBD Medicated Serum on the Expression of JAK2/STAT3 Pathway in HK-2 Cells Induced by AngII

As illustrated in Fig. (**7**), subsequent to AngII induction, there was a substantial activation of the JAK2/STAT3 signaling pathway, evidenced by significant increases in the levels of p-JAK2 and p-STAT3, as well as in the ratios of p-JAK2/JAK2 and p-STAT3/STAT3 (*P* < 0.01). After treatment with JDBD or LPT, a marked reduction in the expression of proteins associated with this pathway was observed (*P* < 0.05 and *P* < 0.01). These findings suggest that the modulatory effects of JDBD on the JAK2/STAT3 pathway are primarily attributed to the inhibition of phosphorylation of the JAK2 and STAT3 proteins.

## 4 DISCUSSION

RIF is the primary pathological factor driving the progression of CKD towards end-stage renal disease and renal failure [2, 26]. The progression of RIF critically influences CKD prognosis; thus, the identification of effective pharmacological strategies to prevent RIF is crucial for CKD management [27, 28]. JDBD, composed of the traditional Chinese medicinal herbs *Astragalus membranaceus*, *Angelica sinensis*, and *Panax ginseng*, has shown promising results. The combined decoction of *Astragalus membranaceus* and *Angelica sinensis* promotes the dissolution of active components, resulting in significantly higher contents of astragaloside IV, calycosin, formononetin, ferulic acid, and ligustilide compared to single herbs or their mixed decoction [29]. Astragaloside IV prevents the progression of RIF by inhibiting inflammation and EMT through the TLR4/NF-κB and Akt/mTOR signaling pathways [30,31]. Ferulic acid alleviates RIF and restores renal function by reducing oxidative stress, inhibiting apoptosis of RTECs, and mitigating kidney damage [32, 33]. Pharmacokinetic studies indicate that when *Astragalus membranaceus* and *Angelica sinensis* are co-administered together, *Angelica sinensis* enhances the bioavailability of astragaloside IV from *Astragalus membranaceus*, accelerating its pharmacodynamic effects and reducing the time to peak plasma concentration by half. In turn, *Astragalus membranaceus* accelerates the absorption rate of ferulic acid from *Angelica sinensis* [34]. Furthermore, studies have confirmed that ligustilide in *Angelica sinensis* can increase the bioavailability of ferulic acid by inhibiting P-glycoprotein [35]. Additionally, ferulic acid promotes the levels of astragaloside IV, calycosin, and formononetin [36]. It is proved that the combination of *Astragalus membranaceus* and *Angelica sinensis* provides a better therapeutic effect, which is in line with the theory of "synergistic enhancement" in Traditional Chinese Medicine. The synergistic application of *Astragalus membranaceus* and *Angelica sinensis* has shown efficacy in preventing renal fibrosis and deterioration of renal function [37-39]. These herbs function by reducing the ECM-associated factors *via* the downregulation of TIMP-1 and TGF-β1 genes, NLRP3 inflammasome, and IL-1β secretion and by enhancing MMP-9 gene expression, thereby mitigating renal fibrosis [40, 41]. *Panax ginseng* is well known for its potent ability to tonify vital energy (Qi). According to the Traditional Chinese Medicine theory that "Qi is the commander of the blood," the addition of *Panax ginseng* to DBD enhances its efficacy in tonifying Qi, promoting blood generation, improving blood circulation, and stabilizing blood. *Panax ginseng* has shown significant potential in combating fibroproliferation. Recent studies suggest that ginseng monomers, active components, and derivatives can inhibit ECM deposition and delay organ fibrosis [42-44]. Ginsenoside-Rg1 has demonstrated the ability to prevent or reverse renal fibrosis [14, 45]. Furthermore, the combined use of *Astragalus membranaceus*, *Angelica sinensis*, and *Panax ginseng* has been shown to enhance the absorption of Ginsenosides in rats, compared with *Panax ginseng* alone [46]. Moreover, *Astragalus membranaceus*, *Angelica sinensis*, and *Panax ginseng* have shown substantial efficacy in regulating the JAK2/STAT3 signaling pathway [47-49]. Nevertheless, few studies have directly examined the effects and mechanisms of JDBD on RIF.

In the investigation of RIF, both the UUO model and the AngII-induced HK-2 cell model are employed to elucidate pathological mechanisms and assess novel therapeutic strategies. These models are broadly acknowledged and utilized for delineating the pathogenesis of RIF [50, 51]. In the UUO model, kidneys display pronounced edema and a paler coloration. Aberrant renal function indicators signify a decline in glomerular filtration capacity. Histological staining demonstrated tubular dilation, cellular atrophy, and edema in the renal tubular, accompanied by fibrotic lesions. Treatment with JDBD markedly alleviates renal edema, enhances renal function, mitigates the severity of renal tubular lesions, and reduces the extent of fibrosis.

The ECM is an intricate network consisting of Col, FN, and polysaccharides that form the basement membrane and facilitate the proliferation and transformation of interstitial fibroblasts into myofibroblasts [52, 53]. ECM has the function of supporting cell structure, regulating cell behavior, and tissue repair [54]. Post-injury, excessive accumulation of ECM rapidly increases, hindering effective renal tissue repair and altering renal structure and function [3, 55]. RTECs transform into myofibroblasts through EMT in RIF. This autonomous proliferation of myofibroblasts promotes the deposition of ECM and aggravates RIF [4, 56]. Our findings demonstrate that, relative to the UUO group, the JDBD intervention results in the upregulation of the epithelial marker E-cadherin protein and mRNA. Conversely, the expression of the myofibroblast marker α-SMA protein and mRNA is downregulated, along with the secretion of ECM-associated pro-fibrotic factors. Studies have confirmed the anti-fibrotic renal protective effects of JDBD.

Apoptosis, a programmed cell death process, is widely acknowledged as a fundamental mechanism for maintaining tissue homeostasis and eliminating damaged cells. However, during RIF, excessive apoptosis of RTECs results in tubular atrophy and hinders renal tissue repair [57-59]. Following the onset of apoptosis, components within the damaged renal cells are released, further activating immune responses and inflammation, thereby exacerbating renal fibrosis [60]. In diverse animal models and pathological specimens of kidney diseases, apoptosis of RTECs is significantly more pronounced in areas with severe interstitial fibrosis [61, 62]. The mitochondrial pathway of apoptosis constitutes an intrinsic route, with the Bcl-2 protein family acting as key regulators [63, 64]. Upon pro-apoptotic signals, the intrinsic apoptosis pathway activates, inducing conformational changes and the translocation of pro-apoptotic proteins such as Bax to the mitochondrial outer membrane, leading to the reduction of MMP and increased permeability and the release of cytochrome C, which activates the apoptosis-executing protein Caspase-3 [65-67]. Therefore, a detailed study of the apoptosis mechanisms in RTECs is an effective means of elucidating the pathological mechanisms of RIF. The *in vitro* assays demonstrated that JDBD effectively counteracted the apoptosis of RTECs that was triggered by Ang II. Cells treated with AngII exhibited markedly increased green fluorescence upon JC-1 staining, indicative of decreased mitochondrial membrane potential and enhanced apoptosis. Following the administration of JDBD, the decrease in green fluorescence and the increase in red fluorescence were observed, along with an increase in mitochondrial membrane potential, suggesting attenuated apoptosis. Moreover, Hoechst 33342 staining revealed significant nuclear condensation and fragmentation in the AngII-treated cells. Caspase-3 activity, additionally assessed through co-staining, was observed to be elevated in conditions of nuclear condensation and fragmentation, corresponding with intensified green fluorescence. Subsequent to JDBD treatment, a decrease in both nuclear condensation and fragmentation and Caspase-3 activity was noted, indicative of reduced apoptosis in HK-2 cells. Furthermore, the study also validates the representative apoptosis-related mRNA. Statistical analysis reveals that JDBD can decrease cell apoptosis in renal cells, upregulate anti-apoptotic mRNA Bcl-2 expression, and downregulate pro-apoptotic mRNA Bax and Caspase 3 expression. Therefore, JDBD can inhibit apoptosis in RTECs.

Recent investigations have shown that the JAK2/STAT3 signaling pathway is important in the development of RIF [68-70]. During the course of RIF, the elevated expression of inflammatory cytokines and transforming growth factors leads to the phosphorylation of JAK2, which subsequently activates STAT3. This activation culminates in the phosphorylation of STAT3 at the Y705 site, facilitating its translocation into the nucleus, where it functions as a transcription factor to regulate the expression of target genes [71-73]. Existing research establishes that the activation of the JAK2/STAT3 pathway is intimately associated with EMT, and the activation of STAT3 promotes the proliferation and activation of fibroblasts and myofibroblasts, thus exacerbating RIF [8, 74]. Research has demonstrated that inhibiting the STAT3 signaling pathway can attenuate the EMT process in RTECs [75-77]. Bax and Bcl-2 are downstream targets of STAT3; in RIF, the activation of STAT3 can induce the changes in Bax and Bcl-2, thereby promoting apoptosis in RTECs [78]. Our research findings demonstrate that JDBD can downregulate the expression of p-JAK2 and p-STAT3, consequently inhibiting the activation of this pathway. This suppression potentially underlies JDBD’s capacity to inhibit EMT and apoptosis in RTECs.

## 5 STUDY LIMITATIONS

**Fig. (8):**
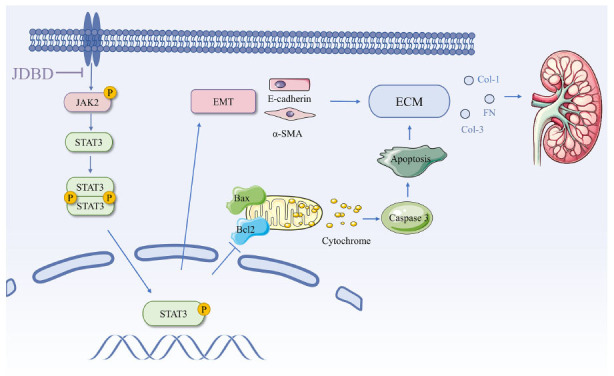
The mechanism map of JDBD reducing RIF by regulating JAK2/STAT3 pathway.

However, this study does have limitations; it did not employ inhibitors specific to the JAK2/STAT3 signaling pathway for verification. This omission makes the relationship between JDBD delaying EMT and inhibiting RTECs apoptosis and the inhibition of the JAK2/STAT3 pathway incompletely reliable. Future studies will combine specific inhibitors to further confirm this relationship. Our research confirms that JDBD can delay EMT and apoptosis in RTECs, thereby decelerating the deposition of the ECM. Furthermore, the JAK2/STAT3 pathway is inhibited during this process, which may represent the pharmacological mechanism through which JDBD combats RIF (Fig. **8**).

## CONCLUSION

In summary, our research findings demonstrate that JDBD can effectively delay the progression of RIF. The probable molecular mechanisms involve the inhibition of the JAK2/STAT3 signaling pathway, which weakens the EMT process and decreases apoptosis in RTECs and ECM deposition. Overall, the traditional Chinese medicine formula JDBD represents a promising therapeutic option for CKD, capable of mitigating renal damage and delaying the progression of RIF.

## Figures and Tables

**Fig. (5):**
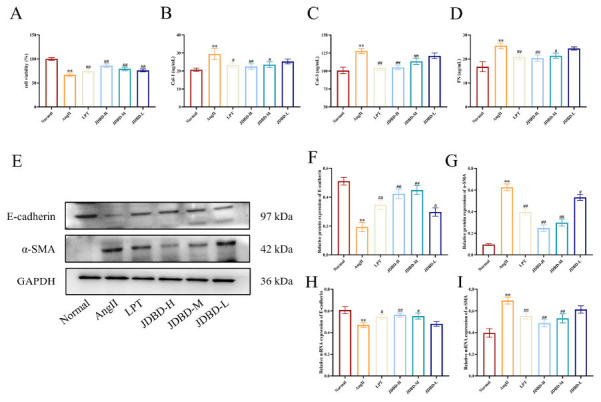
JDBD-containing serum can reduce AngII-induced HK-2 cell viability, ECM deposition and EMT. **(A)** Cell viability was detected by CCK-8 assay. (**B-D**) The levels of Col-1, Col-3 and FN in HK-2 cells supernatant were measured by ELISA. **(E-G)** Western blot analysis showed the levels of E-cadherin and α-SMA protein in HK-2 cells. **(H, I)** qPCR analysis showed the levels of E-cadherin and α-SMA mRNA in HK-2 cells. Data are presented as mean ± SD (*n* = 3). ** *P* < 0.01 *vs.* normal group; ^##^ *P* < 0.01, ^#^ *P* < 0.05 *vs.* AngII group.

**Fig. (7):**
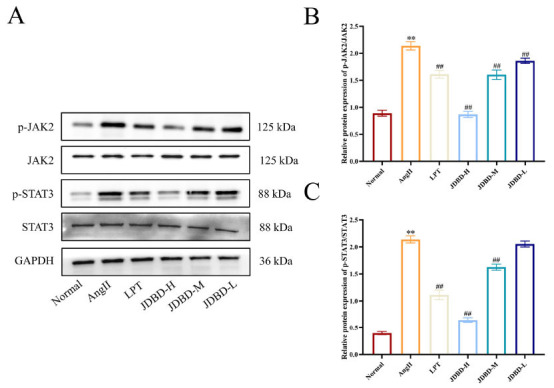
The effect of JDBD medicated serum on the expression of JAK2/STAT3 pathway in HK-2 cells induced by AngII. **(A)** the expression of JAK2/STAT3 pathway-related proteins by Western blot. **(B)** Western blot analysis showed the levels of p-JAK2/JAK2 protein. **(C)** Western blot analysis showed the levels of p-STAT3/STAT3 protein. Data are presented as mean ± SD (*n* = 3). ** *P*< 0.01 *vs.* normal group; ^##^ *P* < 0.01, ^#^ *P* < 0.05 *vs.* AngII group.

**Table 1:** Drug components in each group.

	**Normal**	**AngII**	**LPT**	**JDBD-H**	**JDBD-M**	**JDBD-L**
AngII (10^-6^ mol/L)	-	+	+	+	+	+
JDBD (g/kg)	-	-	-	8.8	4.4	2.2
LPT (10.5 mg/kg)	-	-	+	-	-	-

**Table 2:** The sequence of primers for qPCR.

**Gene name**	**Forward Primer Sequences (5′-3′)**	**Reverse Primer Sequences (5′-3′)**
Bcl-2	ACAGGGTACGATAACCGGGA	CATCCCAGCCTCCGTTATCC
Bax	CATGAAGACAGGGGCCCTTT	CATGAAGACAGGGGCCCTTT
Caspase-3	TGCAGCAAACCTCAGGGAAA	TCTGTTGCCACCTTTCGGTT
E-cadherin	CGAGAGCTACACGTTCACGG	GGGTGTCGAGGGAAAAATAGG
α-SMA	AAAAGACAGCTACGTGGGTGA	GCCATGTTCTATCGGGTACTTC
β‐actin	GTCGTACCACTGGCATTGTG	GTCGTACCACTGGCATTGTG

## Data Availability

The authors confirm that the data supporting the findings of this research are available within the article.

## LIST OF ABBREVIATIONS

AngII: Angiotensin II
Bax: Bcl-2 Associated X
Bcl-2: B-cell Lymphoma-2
BUN: Blood Urea Nitrogen
CCK-8: Cell Counting Kit-8
CKD: Chronic Kidney Disease
Col-1: Col-1 Collagen Types I
DBD: Danggui Buxue Decoction
ECM: Extracellular Matrix
ELISA: Enzyme-linked Immunosorbent Assay
EMT: Epithelial-Mesenchymal Transition
FN: Fibronectin
GAPDH: Glyceraldehyde-3-phosphate dehydrogenase
JAK2: Janus kinase 2
JDBD: Jiawei Danggui Buxue Decoction
LPT: Losartan Potassium Tablets
qPCR: Quantitative Polymerase Chain Reaction
RIF: Renal Interstitial Fibrosis
RTECs: Renal Tubular Epithelial Cells
Scr: Serum Creatinine
STAT3: Signal Transducer and Activator of Transcription 3
UUO: Unilateral Ureteral Obstruction
α-SMA: α-Smooth Muscle Actin

## DECLARATION

There is no content generated by AI in this article. The article only uses the large language model ChatGPT4 to improve the readability of the article.
